# Local and systemic metal ion release occurs intraoperatively during correction and instrumented spinal fusion for scoliosis

**DOI:** 10.1007/s11832-015-0631-6

**Published:** 2015-01-15

**Authors:** William J. Cundy, Annika R. Mascarenhas, Georgia Antoniou, Brian J. C. Freeman, Peter J. Cundy

**Affiliations:** 1 Department of Orthopaedic Surgery, Women’s and Children’s Hospital, 72 King William Road, North Adelaide, SA 5006 Australia; 2 Discipline of Orthopaedics and Trauma, University of Adelaide, Adelaide, SA Australia

**Keywords:** Spinal deformity, Pediatric, Metal, Wear, Titanium

## Abstract

**Study design:**

Prospective pilot study.

**Objectives:**

The aim of this study was to measure titanium, niobium and aluminium levels in various intraoperative and postoperative samples to determine patterns of metal ion release that occur within the first month following instrumented spinal fusion.

**Summary of background data:**

Raised serum metal ion levels are reported following instrumented spinal fusion in adolescent idiopathic scoliosis. The exact topological origin and chronology of metal ion release remains conjectural. Recent literature suggests an immediate rise in serum metal levels within the first postoperative week.

**Methods:**

Titanium, niobium and aluminium levels were measured before, during and after surgery in serum and local intraoperative fluid samples obtained from two pediatric patients undergoing posterior correction and instrumentation for scoliosis.

**Results:**

Measurable metal ion levels were detected in all local samples obtained from wound irrigation fluid, cell saver blood, and fluid that immersed metal universal reduction screw tabs. Postoperative serum metal ion levels were elevated compared to baseline preoperative levels. In general, metal ion levels were considerably higher in the intraoperative fluid samples compared to those observed in the serum levels.

**Conclusion:**

Our findings of contextually high metal ion concentrations in intraoperative and early postoperative samples provide further empirical support of a ‘putting-in’ phenomenon of metal ion release following instrumented spinal fusion. This challenges existing beliefs that metal ion release occurs during an intermediate ‘wearing-in’ phase. We recommend thorough irrigation of the operative site prior to wound closure to dilute and remove intraoperative metal ion debris. Possibilities of filtering trace metal ions from cell saver content may be considered.

## Introduction

Instrumented spinal arthrodesis is the principal surgical treatment for pediatric scoliosis and allows effective, life-long deformity correction. As a result, there have been numerous studies documenting raised serum metal ion levels in this setting [1–4]. It is reported that intracellular phagocytosis of particulate metal debris triggers the release of pro-inflammatory cytokines, generating a cyclical process of chronic inflammation in affected peri-implant tissues. Clinical consequences of this process range from delayed establishment of spinal fusion to complications of osteolysis, pseudarthrosis, aseptic loosening and implant failure [5–10]. There have been few subsequent studies assessing the short-, medium- and long-term sequelae of implant-derived systemic metal dissemination.

Existing studies describe a time-dependent direct relationship between serum metal ion levels and the time since surgery [1–4]. Recent literature demonstrates a chronological systemic release pattern of titanium, niobium and aluminium from spinal implants over the 2-year postoperative period [1]. The data indicate an immediate initial peak in serum metal levels within the early postoperative period, with a gradual decline and an apparent plateau thereafter. These findings challenge previous understanding of metal ion release in that the rapid rise in serum titanium and niobium levels observed within the first postoperative week are more consistent with a ‘putting-in’ rather than a ‘wearing-in’ phenomenon. The origin of the early metal ion release is unknown and has prompted this pilot study to explore the intraoperative pattern of metal ion release.

## Methods and materials

This study was approved by the Women’s and Children’s Hospital, Children Youth and Women’s Health Service Human Research Ethics Committee (REC2084/7/11). Two patients enrolled in an ongoing prospective longitudinal study were recruited and completed standardized questionnaires to enquire about possible environmental exposure sources to titanium, niobium and aluminum. Patients with pre-existing metallic implants in situ were excluded.

Both patients underwent standard posterior instrumented correction and fusion of thoracic vertebrae (T4 to T12) for adolescent idiopathic scoliosis using the Universal Reduction Screw (URS) System (DePuy Synthes Spine, Synthes GmbH, Oberdorf, Switzerland). The URS system (2nd generation) is comprised of mixed phase alpha–beta titanium-based alloy. The metal alloy composition of this implant is approximately 87 % titanium, 6 % aluminium and 7 % niobium. Operative field blood was collected by suction and delivered by plastic tubing to an autotransfusion cell salvage machine (Sorin Electa Compact Autotransfusion Device, Sorin Group, CO, USA). Autologous blood collected in this way is routinely returned to the patient perioperatively.

Three intraoperative samples were collected from each patient. Sample 1 was taken immediately following insertion of precontoured rods and securing the rods to pedicle screws, but prior to wound closure. The wound bed was irrigated with 250 ml of 0.9 % normal saline and allowed to sit for 2 min exactly. A 10-ml sample of the fluid was then aspirated and immediately transferred into 5-ml trace element-free Vacutainer^®^ tubes (Becton, Dickinson and Company, NJ, USA). This specimen was labeled ‘wound irrigation’.

Sample 2 was taken following routine removal of the reduction tabs at the completion of the posterior construct once all screws had been tightened. The tabs were placed in a sterile bowl and covered with 10 ml sterile 0.9 % normal saline. After 5 min exactly, 5 ml was aspirated and transferred into a trace element-free Vacutainer^®^ tube. This specimen was labeled ‘tab washings’.

A 5-ml sample (sample 3) was taken from the washed and prepared cell saver blood prior to return of this autologous blood to the patient. This specimen was labeled ‘cell saver’.

In addition, both patients underwent serum blood sampling at the time of induction of general anesthesia (sample 4), as well as at three different time intervals postoperatively—postoperative day 1 (sample 5), postoperative day 7 (sample 6), and postoperative day 28 (sample 7).

These samples were collected and stored according to contamination-free consensus guidelines. Venepuncture was performed using a stainless steel needle (not containing titanium, niobium or aluminium) into trace element-free Vacutainer^®^ tubes. Intraoperative washings were again collected with sterile syringes free of the investigated metal ions. Samples were collected and transported for analysis of metal concentration using high-resolution inductively coupled plasma mass spectrometry at a commercial laboratory (ALS Scandinavia AB, Lulea, Sweden). Limit of detection for titanium, niobium and aluminium was 0.1, 0.02 and 0.2 parts per billion, respectively. The reference range of metal ion levels detected from the preoperative blood samples were expected to reflect those of the local population.

## Results

### Intraoperative metal ion levels

**Fig. 1:**
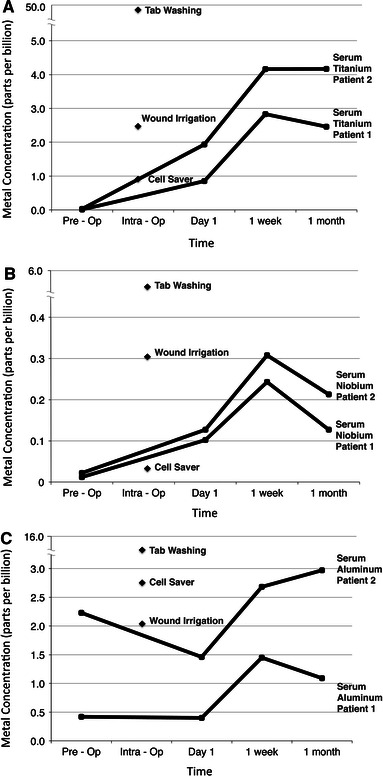
Titanium, niobium and aluminium levels from local and systemic fluid samples taken at various time intervals (Fig. 1a, b, c, respectively). Cell saver and wound irrigation data *labels* represent mean values (*n* = 2)

Raised titanium, niobium and aluminium levels were recorded in the wound irrigation fluid (Fig. 1a, b, c). With the exception of titanium, wound irrigation levels were higher than all serum metal ion samples.

The highest concentrations of all three metal ions were recorded from the intraoperative tab washings.

Low but detectable levels of metal ions were observed in the cell saver samples, despite the suctioned blood being cleaned, centrifuged and filtered before being removed for analysis.

### Serum metal ion levels

Preoperative titanium and niobium levels were low as expected; however, preoperative aluminium serum level was elevated and variable between the samples studied.

An immediate postoperative rise in serum titanium and niobium metal ion levels was observed compared to the preoperative baseline levels (Fig. 1a, b). These serum metal ion levels rose most abruptly between the first postoperative day and 1 week postoperatively.

## Discussion

Spinal arthrodesis with titanium alloy instrumentation is popular due to higher corrosion resistance and biocompatibility in comparison with other biomaterials such as stainless steel and cobalt chrome alloys. There is current literature that provides concerning evidence to support metal wear debris being systemically distributed and deposited in solid organs. This literature is confined to animal model and human cadaveric studies only and relates exclusively to hip replacement prostheses [11, 12]. A number of human studies investigating serum metal ion levels following spinal instrumentation (both stainless steel and titanium alloys) all report abnormally elevated metal ion levels (chromium, nickel, iron, titanium and aluminium) [10, 13–18]. Wang et al. demonstrated that patients with pseudarthrosis of titanium spinal implants generated much higher metal debris [6]. On the basis of these findings, it seems reasonable to suggest a self-perpetuating cycle of wear, corrosion and implant failure in spinal implants contributing to these findings. However, the exact moment of release of metal ions during the process has not been extensively reviewed or studied.

The detection of metal ions in wound bed irrigation fluid provides compelling evidence to suggest that metal ions are released at the time of surgery. It is suggested that these metal ions are released during the necessary handling of instrumentation, the mechanical stresses applied during tightening of instrumentation and/or the interaction with exposed metal surfaces, particularly when snapping off reduction tabs in these cases. Together with emerging evidence from longitudinal studies involving serum metal measurements, these findings consolidate the hypothesis that a ‘putting-in’ phenomenon of metal ion release occurs. As the serum metal ion levels continued to remain elevated, or elevate further at 1 week and 1 month postoperatively, this would suggest that release of metal ions might also be an ongoing process while ‘wearing in’ of the construct occurs.

Although most of the wound bed irrigation fluid is suctioned before wound closure, it is inevitable that a small residual volume remains that would either deposit trace metal ions locally, or enter the circulatory volume for systemic distribution. The detection of raised serum metal ion levels in the first postoperative week would support the latter. It is routine at our institution not to insert wound drains and therefore, in the absence of any leakage from the wound, metal ions within operative site hematoma or inflammatory fluid must be resorbed.

The cell saver blood collected throughout surgery included blood loss during the initial dissection and exposure, blood loss during pedicle screw and rod insertion, as well as blood loss from the operative site during decortication, facet joint excision and up to the time of wound closure. Therefore, the cell saver blood was an aggregate of blood that may or may not have been exposed to the metal implants. The non-physiologic levels of metal ions detected in cell saver blood indicate that a metal ion ‘load’ is returned to the patient with the autologous transfusion.

The very high metal ion levels seen in reduction screw tab washings suggest that this exposed unoxidized metal surface (at the point where pedicle screw tabs are broken off) might be a more active site of metal ion release. This surface is unpassivated, whereas the unaltered surface of the remaining construct is passivated. This exposed area of unpassivated metal is not dissimilar to the area exposed by the threads and torqued compressions between the rod and the pedicle screws or cross connectors. It appears likely that the maximal exposure of metal ions would occur with exposed unpassivated metal interfaces as well as with abrading of the anodized surfaces during the torquing of nuts and caps.

Our study noted that preoperative metal ion levels for titanium and niobium were universally low. However, in stark contrast, we found a variation in the preoperative ‘normal baseline’ serum aluminium levels, which was also shown in earlier studies by Cundy et al. [1, 3]. Environmental exposure to aluminium may account for this wide variance given the countless sources of daily exposure and this may account for preoperative and postoperative elevated measurements, given the inability to regulate this exposure. We suspect this recognized source of uncontrollable error prevents the determination of a true value and therefore we cannot exclude the possibility of an elevated postoperative burden of aluminium.

The limitations of the study include a small study population (*n* = 2), arbitrary volumes of normal saline irrigation fluid, and inconsistent duration that the test fluid was allowed to sit in the operative field prior to sampling.

## Conclusions

This study demonstrates measurable metal ion levels in intraoperative fluid samples, indicating that metal debris is present in the operative field. This implicates metal ion release at the time of implantation and further supports a ‘putting in’ phenomenon rather than, or in addition to a ‘wearing in’ phenomenon of metal ion release. The finding of low but detectable metal ion levels in cell-salvaged blood could increase metal ion burden to the patient when transfused perioperatively. It is possible that thorough irrigation of the operative site prior to closure may both dilute and remove local metal ions, thereby reducing the overall metal ion burden to the patient. This study investigated a specific spinal implant utilized in both patients. Hence, further studies employing other implant designs and interfaces may provide clues to identify and understand the exact locations of metal ion release and its chronological pattern.
